# Protein Fraction and Derived Peptides from *Amaranthus hypochondriacus*: A Preliminary In Vitro Study of Cytotoxic Activity in Cancer Cell Lines

**DOI:** 10.3390/ijms27114900

**Published:** 2026-05-28

**Authors:** Fernanda Reséndiz-Otero, Carmen Valadez-Vega, Belinda Patricia Velázquez-Morales, Aurea Bernardino-Nicanor, Raúl Velasco-Azorsa, Gabriel Betanzos-Cabrera, Arturo Salazar-Campos, Víctor Manuel Muñoz-Pérez, José Antonio Morales-González

**Affiliations:** 1 Área Académica de Medicina, Instituto de Ciencias de la Salud, Universidad Autónoma del Estado de Hidalgo, Ex-Hacienda de la Concepción, San Agustín Tlaxiaca 42080, Hidalgo, Mexico; mafernanda.resendiz@gmail.com (F.R.-O.); l.n.belindavelmor@gmail.com (B.P.V.-M.); arturo_salazar10347@uaeh.edu.mx (A.S.-C.); victor9673@hotmail.com (V.M.M.-P.); 2 Tecnológico Nacional de Mexico/IT Celaya, Antonio García Cubas Pte #600 esq. Av. Tecnológico, Celaya 38010, Guanajuato, Mexico; aurea.bernardino@itcelaya.edu.mx; 3 Área Académica de Biología, Instituto de Ciencias Básicas e Ingeniería, Universidad Autónoma del Estado de Hidalgo, Mineral de la Reforma 42184, Hidalgo, Mexico; raul_velasco@uaeh.edu.mx; 4 Área Académica de Nutrición, Instituto de Ciencias de la Salud, Universidad Autónoma del Estado de Hidalgo, Ex-Hacienda de la Concepción, San Agustín Tlaxiaca 42080, Hidalgo, Mexico; gbetanzo@uaeh.edu.mx; 5 Laboratorio de Medicina de Conservación, Escuela Superior de Medicina, Instituto Politécnico Nacional, Plan de San Luis y Díaz Mirón, Ciudad de México 11340, Del. Miguel Hidalgo, Mexico; jmorales101@yahoo.com.mx

**Keywords:** breast cancer, cervical cancer, amaranth proteins, amaranth peptides, cytotoxicity

## Abstract

Amaranth proteins have been associated with various health benefits, including anticancer activity. This study aimed to evaluate the cytotoxic effect of proteins and peptides derived from *Amaranthus hypochondriacus*. A crude extract (CE) and protein fraction (PF) were obtained; peptides were produced by enzymatic hydrolysis, yielding one sample > 30 kDa (P > 30 kDa) and another < 30 kDa (P < 30 kDa). Protein, phenolic, and antinutritional compound content were determined, along with antioxidant capacity and cytotoxic activity against MDA-MB-231 and SiHa cells. CE exhibited the highest protein content of 182.6 mg/g, total phenols 129.40 mg GAE/g, and antioxidant capacity by both methods, 104.50 µM TEAC/g for ABTS•+ and 75.62 µM TEAC/g for DPPH•. PF showed the greatest concentration of saponins and lectins, 80 HU/mg and 60.30 HA/mg, respectively. PF displayed the strongest cytotoxic effect at 72 h in both cell lines, with IC_50_ of 44.98 µg/mL for MDA-MB-231 cells and 69.24 µg/mL for SiHa cell line. Transmission electron microscopy (TEM) revealed severe structural damage induced by PF in both cell lines. In conclusion, PF from *Amaranthus hypochondriacus* seeds showed the greatest in vitro cytotoxic effect against both cancer cell lines, principally in MDA-MB-231 cells.

## 1. Introduction

Cancer remains one of the leading causes of morbidity and mortality worldwide, accounting for 9,743,832 deaths in 2022. In women, breast and cervical cancers are among the most prevalent types. These malignancies are characterized by abnormal cell growth resulting from a limited number of genetic mutations, either inherited or induced by external factors [1,2]. Despite advances in diagnosis and treatment, there is still a continuous need for novel bioactive compounds that may contribute to complementary strategies in cancer-related research, particularly those derived from natural sources [3].

Chronic diseases such as cancer may have an important nutritional component, implying that dietary patterns can either increase the risk of development and progression or, conversely, provide protective effects by reducing disease incidence and supporting treatment. Therefore, maintaining healthy eating habits and incorporating functional foods are essential strategies that may help reduce or even prevent the onset of various chronic diseases [4].

Scientific reports have shown that regular consumption of functional foods, such as pseudocereals, may reduce the risk of developing various chronic diseases. Among these foods, amaranth (*Amaranthus* spp.) has attracted considerable attention due to its high nutritional quality, balanced amino acid profile, high protein digestibility, and ability to grow under adverse climate and soil conditions [5,6,7].

In addition to improving human nutrition, amaranth proteins and derived peptides have demonstrated important functional and biological properties, including antioxidant, antihypertensive, and antiproliferative activities, suggesting their potential role in the prevention or treatment of chronic diseases, including cancer [8,9].

Consequently, the incorporation of plant-derived proteins into food products has become increasingly common to enhance nutritional quality and provide essential amino acids required for maintaining good health. Owing to their valuable nutritional and functional characteristics, amaranth proteins have been widely explored in both native and modified forms for the development of enriched and functional food products [10,11].

In recent years, plant-derived proteins and peptides have gained increasing attention due to their diverse biological activities, including antioxidant, antihypertensive, immunomodulatory, and cytotoxic effects, which are associated with their ability to modulate cellular processes such as oxidative stress, cell cycle progression, and apoptosis. These properties are closely related to structural characteristics such as amino acid composition, sequence, and molecular size, as well as to processing conditions employed during their preparation [12,13,14,15].

The generation of bioactive peptides through enzymatic hydrolysis of proteins has been explored as an effective strategy to enhance biological activity. It has been reported that the source of protein, the type of enzymes used, hydrolysis conditions, and the resulting peptide size distribution play a critical role in determining the functional properties and bioactivity of the resulting peptides [12,16,17].

However, despite these reports, information regarding the cytotoxic effects of protein fractions and enzymatically derived peptides from *Amaranthus hypochondriacus* remains limited, particularly in terms of comparative evaluation between protein fractions and their hydrolysates, as well as the associated cellular morphological effects. Therefore, the aim of the present study was to conduct a preliminary evaluation of the in vitro cytotoxic activity of protein fraction and derived peptides from *Amaranthus hypochondriacus* against breast (MDA-MB-231) and cervical (SiHa) cancer cell lines and to assess the associated cellular damage through ultrastructural analysis using transmission electron microscopy, in order to provide foundational information for future studies focused on the identification and characterization of bioactive peptides from amaranth proteins.

## 2. Results

### 2.1. Obtained Samples and Degree of Hydrolysis of Protein Fraction

The obtained samples included the crude extract (CE) and the protein fraction (PF). Regarding the degree of hydrolysis (DH), enzymatic hydrolysis of PF produced a high proportion (99%) of trichloroacetic acid (TCA)-soluble nitrogenous compounds according to the applied method, indicating extensive proteolysis under the experimental conditions. This process yielded two peptide samples, one with molecular weight > 30 kDa (P > 30 kDa) and another with molecular weight < 30 kDa (P < 30 kDa).

### 2.2. Protein Content

**Figure 1:**
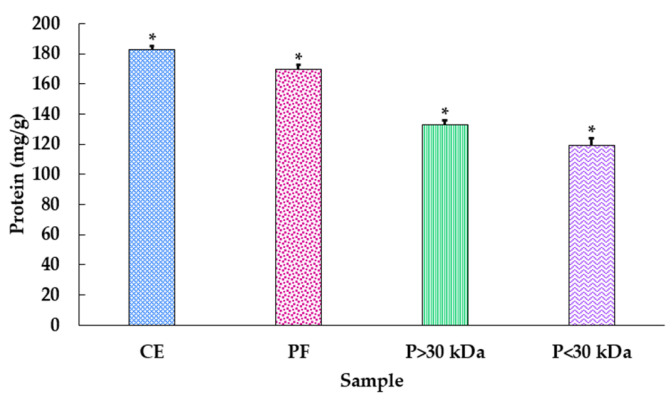
Protein concentration in amaranth samples. Crude extract (CE), protein fraction (PF), peptides > 30 kDa (P > 30 kDa), peptides < 30 kDa (P < 30 kDa). Bars represent the mean ± standard deviation (SD) of three independent experiments. Asterisks indicate statistically significant difference according to Tukey post hoc test (*p* ≤ 0.05).

As shown in Figure 1, the protein content of the analyzed samples decreased after CE extraction process, showing a reduction of 7% in PF (*p* < 0.05). Enzymatic hydrolysis of PF significantly decreased the protein content (*p* < 0.05), with reductions of 21.8 and 29.7% in P > 30 kDa and P < 30 kDa, respectively.

### 2.3. Phenolic and Antinutritional Compound Content

Table 1 shows the phenolic and antinutritional compound content in the amaranth samples. Regarding total phenols, significantly higher content (*p* ≤ 0.05) was observed in CE (78.35%) compared to PF, with a marked decrease in the presence of these compounds in the peptide samples (*p* ≤ 0.05).

Saponins were detected only in CE and PF, showing the highest content in PF (*p* ≤ 0.05), these metabolites were absent in both peptide samples. The highest hemagglutination activity was observed in PF, showing a statistically significant difference (*p* ≤ 0.05) compared to CE, lectins were not detected in peptide samples.

### 2.4. Antioxidant Capacity of Amaranth Samples

The results shown in Table 2 indicate significant differences between the four samples evaluated (*p* ≤ 0.05). CE exhibited the highest antioxidant capacity, with a progressive decrease observed throughout the peptide extraction process in both methods. The 2,2′-Azino-bis(3-ethylbenzothiazoline-6-sulfonic acid) (ABTS•+) assay yielded higher values than the 2,2-diphenyl-1-picrylhydrazyl radical (DPPH•) assay. According to the ABTS•+ results, the antioxidant capacity of PF was 81.4% lower than CE (*p* ≤ 0.05), whereas in P < 30 kDa, a 92.3% decrease was observed (*p* ≤ 0.05). Observing that peptides with lower molecular weight showed lower antioxidant capacity. In contrast, based on the DPPH• assay, the antioxidant capacity of PF and P > 30 kDa decreased by 77.38% and 87.54%, respectively, compared with CE (*p* ≤ 0.05).

In the ABTS•+ assay, the mean inhibitory concentration (IC_50_) value was highest in P > 30 kDa, being 94.79-fold higher than that of the P < 30 kDa (*p* ≤ 0.05), while CE exhibited the lowest value (*p* ≤ 0.05). A similar trend was observed in the DPPH• assay, with P > 30 kDa and P < 30 kDa showing higher IC_50_ values compared to CE and PF (*p* ≤ 0.05). The IC_50_ of CE was 96.88-fold lower than that of P > 30 kDa (*p* ≤ 0.05).

### 2.5. Cytotoxicity of Amaranth Samples on MDA-MB-231 and SiHa Cells

**Figure 2:**
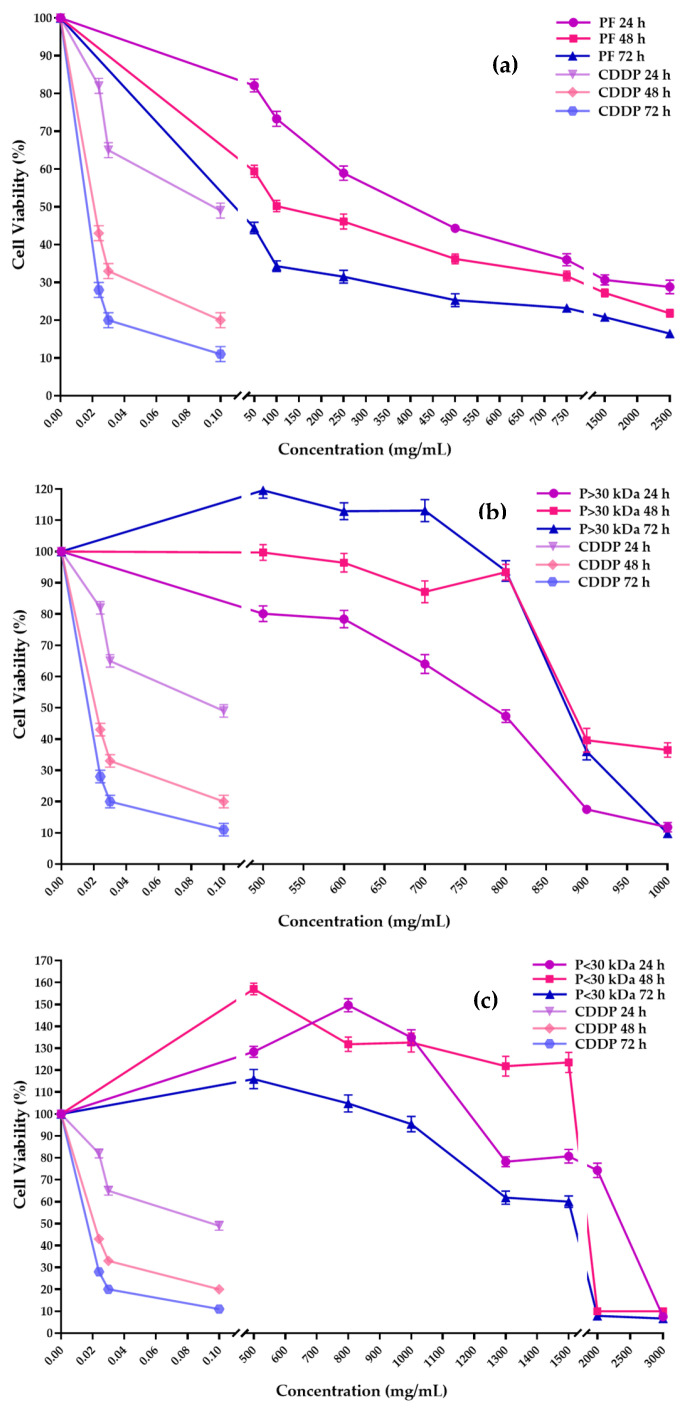
Cytotoxic effect of *Amaranthus hypochondriacus* samples on MDA-MB-231 cells at 24, 48 and 72 h. (**a**) Protein fraction (PF), (**b**) peptides > 30 kDa (P > 30 kDa), and (**c**) peptides < 30 kDa (P < 30 kDa). Cisplatin (CDDP). Points represent the mean ± SD of three independent experiments.

Figure 2 presents the cytotoxic effects of PF, P < 30 kDa and P > 30 kDa on the MDA-MB-231 cell line. As shown in Figure 2a, exposure to PF resulted in a significant (*p* ≤ 0.05) time and dose-dependent decrease in cell viability, with the greatest reduction observed after 72 h of exposure (*p* ≤ 0.05). At a concentration of 2500 µg/mL, cell viability was inhibited by 83.6%, a value that was statistically different from those at 24 and 48 h (*p* ≤ 0.05).

For P > 30 kDa (Figure 2b), cell viability decreased at 24 and 48 h, showing an apparent dose-dependent effect. The reduction was most pronounced at 24 h, when viability was inhibited by 88.2% at 1000 µg/mL (*p* ≤ 0.05), whereas after 48 h, inhibition at the same concentration reached 63.5% (*p* ≤ 0.05). After 72 h of exposure, cell viability increased by 13–19% within the 500–700 µg/mL range, and then decreased at concentrations above 800 µg/mL, reaching 9.8% at 1000 µg/mL (*p* ≤ 0.05).

For P < 30 kDa (Figure 2c), an increase in cell viability was observed at concentrations between 500 and 1500 µg/mL. Compared with the control, cell viability increased by 49, 57, and 15.9% at 24, 48, and 72 h, respectively. At the highest concentration tested (3000 µg/mL), a significant decrease in viability was observed, with values of 7.5, 11, and 6.7% at 24, 48, and 72 h, respectively (*p* ≤ 0.05).

Cisplatin (CDDP) exhibited a time- and dose-dependent cytotoxic effect and showed greater cytotoxicity compared with all amaranth-derived samples (Figure 2).

**Figure 3:**
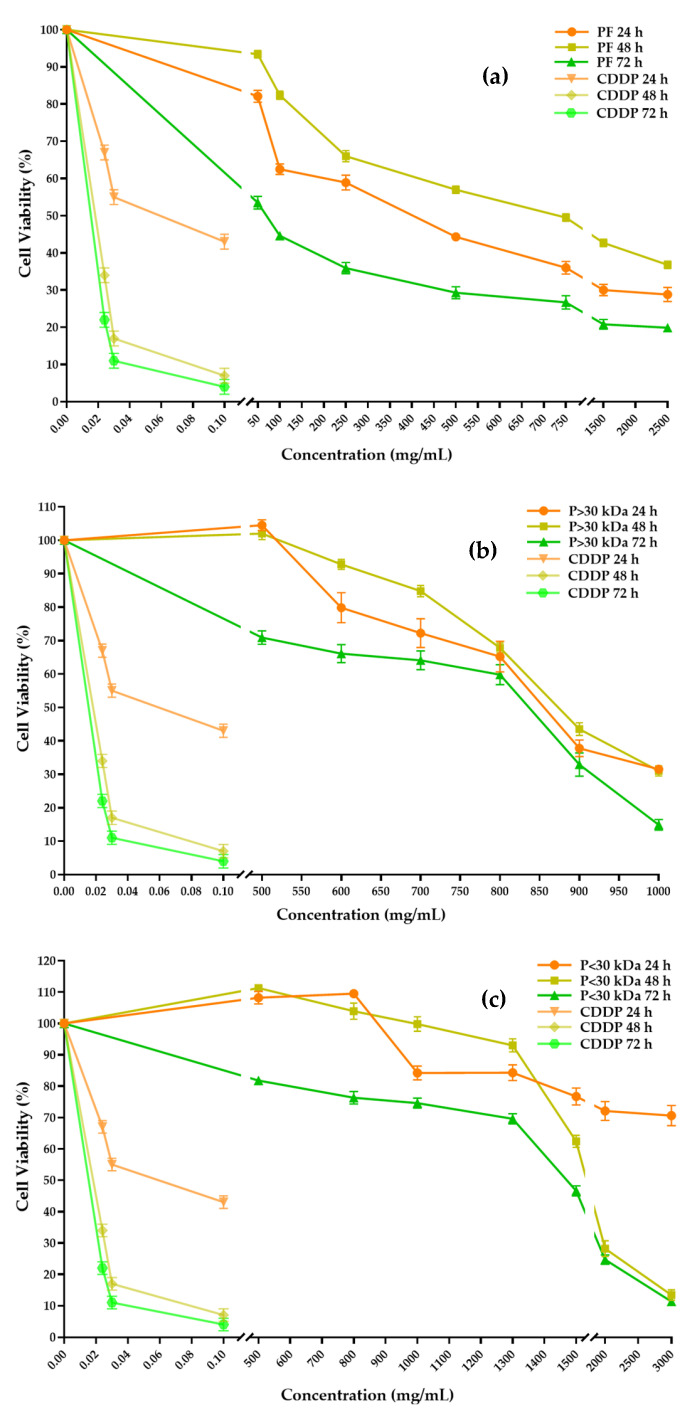
Cytotoxic effect of *Amaranthus hypochondriacus* samples on SiHa cells at 24, 48 and 72 h. (**a**) Protein fraction (PF), (**b**) peptides > 30 kDa (P > 30 kDa), and (**c**) peptides < 30 kDa (P < 30 kDa). Cisplatin (CDDP). Points represent the mean ± SD of three independent experiments.

The results of the cytotoxicity assay on SiHa cells are presented in Figure 3. As shown in Figure 3a, treatment with PF exhibited a clear dose-dependent effect (Figure 3a). After 24 h of treatment, cell viability decreased significantly (*p* ≤ 0.05), ranging from 18 to 71.2%. Treatment for 48 h resulted in 6.6–63.2% inhibition of cell viability (*p* ≤ 0.05). The most pronounced effect was observed after 72 h, when cell viability decreased by 46.5–80.1% (*p* ≤ 0.05).

P > 30 kDa (Figure 3b) induced a reduction in cell viability after 24 h of treatment, with inhibition beginning at 600 µg/mL (*p* ≤ 0.05). After 48 h, these peptides exhibited lower cytotoxicity (*p* ≤ 0.05). The most pronounced effect was observed after 72 h, when cell viability decreased by 29.1–85.1% (*p* ≤ 0.05).

P < 30 kDa (Figure 3c) increased cell viability (3.9–11.3%) at 24 and 48 h at concentrations of 500 and 800 µg/mL. However, at higher concentrations, a reduction in viability was observed, reaching 29.4% at 24 h and 86.6% at 48 h (*p* ≤ 0.05). The strongest cytotoxic effect was detected after 72 h of exposure, with inhibition ranging from 18.3 to 88.7% (*p* ≤ 0.05).

Figure 3 shows that CDDP exhibited greater cytotoxic activity than the amaranth samples at all exposure times.

The IC_50_ values obtained from the cytotoxicity assays are summarized in Table 3. In MDA-MB-231 cells, a time-dependent cytotoxic effect was observed, with PF showing the highest bioactivity at 72 h (*p* ≤ 0.05). Similarly, in SiHa cells, all samples exhibited their greatest effect at 72 h, with PF demonstrating the highest cytotoxicity (*p* ≤ 0.05). PF exhibited the highest cytotoxicity in both cell lines; therefore, it was selected for further analysis by TEM.

### 2.6. Transmission Electron Microscopy

**Figure 4:**
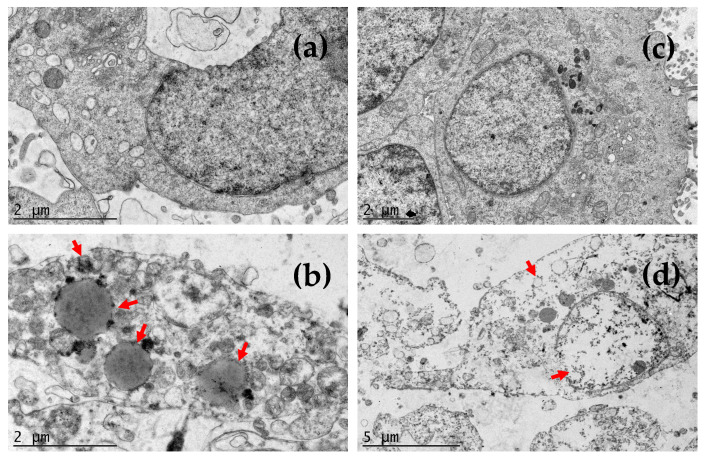
Transmission electron microscopy (TEM) images (**a**) MDA-MB-231 control cells cultured in Dulbecco’s modified Eagle’s medium (DMEM) supplemented with fetal bovine serum (FBS), (**b**) MDA-MB-231 cells treated with protein fraction (PF; 44.98 µg/mL) for 72 h showed the formation of vacuoles (arrows), (**c**) SiHa control cells cultured in DMEM supplemented with FBS, and (**d**) SiHa cells treated with PF (69.24 µg/mL) for 72 h showed vacuoles formation, membrane disruption, and loss of cell organelles (arrows). Experiments were carried out in triplicate.

TEM images of MDA-MB-231 and SiHa cells are shown in Figure 4. In the MDA-MB-231 control group (Figure 4a), cells cultured in Dulbecco’s modified Eagle’s medium (DMEM) supplemented with fetal bovine serum (FBS), retained their normal morphology after 72 h of culture. In contrast, MDA-MB-231 cells treated with PF (44.98 µg/mL) for 72 h (Figure 4b) exhibited severe structural damage, characterized by vacuolation and loss of mitochondrial, nuclear, and cell plasma membrane integrity.

In the SiHa control group (Figure 4c), cells cultured in DMEM supplemented with FBS maintained their structural integrity. In contrast, SiHa cells treated with PF (69.24 µg/mL) for 72 h (Figure 4d) displayed severe ultrastructural damage, including disruption of the cell plasma membrane and loss of organelles such as the nucleus and mitochondria.

## 3. Discussion

The total protein content of the amaranth samples evaluated in the present study was comparable with that reported by other authors, ranging from 11.35 to 18.8% [18,19], supporting the consistency of our results with existing literature. However, the broader range reported for species such as *Amaranthus cruentus*, *Amaranthus caudatus*, *Amaranthus hybridus*, *Amaranthus albus*, and other *Amaranthus* species (12.5 to 33.5%), suggests that protein levels are dependent on the species, cultivation conditions and environmental factors [18,20]. Such variations in protein content could influence the nutritional, functional and biological properties, including antioxidant and cytotoxic activities.

The protein content of amaranth seeds may vary depending on genotype, environmental conditions, and analytical methods. The protein values reported in the present study were comparable to those reported for other pseudocereals such as buckwheat, quinoa, and chia, which show protein ranges of 12.8–13.27%, 10–18%, and 15–26.03%, respectively. These ranges can partially coincide among species; therefore, comparisons should be interpreted considering this variability. In contrast, when compared with common cereals such as rice, corn, and rye, amaranth generally tends to show higher protein content, as these cereals typically contain between 7 and 13% protein [21].

Due to their high protein content and favorable amino acid profile, amaranth seeds have been recommended for human consumption. In addition, they represent a potential source of raw material to produce protein concentrates, protein-rich fractions or purified proteins [21].

In many parts of the world, the use of plant-based proteins as substitutes for animal proteins has increased, primarily due to their lower cost. From a nutritional standpoint, plant proteins, such as those derived from amaranth, provide the essential amino acids required for human health [22]. Therefore, obtaining protein concentrates from amaranth is of great importance, as they have the potential to be used in the formulation of high-protein food supplements. Furthermore, these protein concentrates can supply amino acids and bioactive peptides released during digestion, which may exert important biological effects, including antioxidant, antihypertensive, anti-inflammatory, and antitumor activities [23].

The protein content of CE obtained in this study was 182.6 mg/g, which is higher than the values reported by other authors for *Amaranthus cruentus* (90.5–194.4 mg/g), *Amaranthus leucocarpus* (81.2 mg/g), *Amaranthus cruentus* (142.5 mg/g), and *Amaranthus spinosus* (108 mg/g), but similar to those of *Amaranthus caudatus* (186.5 mg/g) [24,25]. These results indicate that the conditions used for obtaining CE were suitable for achieving efficient protein recovery.

It has been reported that the conditions used for obtaining PF favor the extraction of globulins, which are among the most abundant proteins in amaranth seeds. This group of proteins encompasses several bioactive proteins and peptides, including enzymes, lectins, and enzyme inhibitors, among others [26,27].

Proteins obtained from several sources can be modified through enzymatic hydrolysis to produce peptides that have attracted considerable attention due to their potential biological functions, including antihypertensive, anti-inflammatory, antithrombotic, and antioxidant activities [28].

Through enzymatic hydrolysis of PF from *Amaranthus hypochondriacus,* a 99% DH was achieved. This result was higher than that reported by Montoya-Rodríguez [29], who obtained 31% hydrolysis in the same amaranth species. The hydrolysis process applied to *Amaranthus hypochondriacus* proteins also proved to be more effective than that reported for proteins from *Phaseolus coccineus* and Glycine max using the same enzymes, in which 73% hydrolysis was achieved [30,31].

It has been reported that DH depends on the type of proteins used and the specificity of the enzymes. Furthermore, the number of peptides generated can be increased by employing combinations of different enzymes [32,33].

CE exhibited the highest antioxidant activity, followed by PF and peptide samples. It was also observed that CE and PF required lower concentrations to inhibit 50% of the oxidizing agents (ABTS•+ and DPPH•). In contrast, the two peptide samples were not effective in scavenging the radicals. These results are consistent with previous reports indicating that protein hydrolysis may have an adverse effect on antioxidant capacity, as this process may cause partial or total loss of activity. In some cases, specific peptides formed may exhibit pro-oxidant activity, counteracting the antioxidant effects of other peptides [34]. This was corroborated in the present study, as enzymatic hydrolysis significantly reduced the antioxidant capacity compared to CE and PF.

Previous studies indicated that the amino acid composition plays an important role in the antioxidant capacity of peptides, which tends to increase in the presence of hydrophobic and aromatic amino acids [35]. Amino acids such as tyrosine, methionine, histidine, lysine, tryptophan, and proline, in their free form, have been shown to exhibit antioxidant activity by other authors [36]. Although the presence of these amino acids is important for antioxidant activity, Najafian et al. [37] reported that the sequence in which they occur within peptides is also critical, as it contributes to an enhanced antioxidant capacity.

In the present study, although both peptides exhibited low antioxidant activity, P < 30 kDa showed higher activity than P > 30 kDa in the DPPH• assay. This finding is consistent with previous studies, which indicate that the antioxidant capacity of peptides is related to their molecular weight, with lower molecular weight peptides generally exhibiting higher antioxidant activity. Furthermore, factors such as amino acid sequence and composition, the structure of the precursor proteins, and the hydrolysis process used to obtain the peptides also influence their antioxidant activity [38,39].

Ayala-Niño et al. [40] reported the antioxidant capacity of proteins from *Amaranthus hypochondriacus* and their corresponding peptides obtained by hydrolysis with alcalase and flavourzyme, showing values lower than those obtained in the present study. Similarly, Orsini et al. [41] reported lower values in peptides derived from *Amaranthus mantegazzianus* proteins hydrolyzed with pepsin and pancreatin. These results are consistent with the previous discussion, as factors such as the protein source, the extraction process, and the hydrolysis method play a significant role in determining antioxidant capacity.

Differences in the antioxidant capacity of albumins and proteins from *Amaranthus retroflexus* have been reported by other authors, with albumins exhibiting higher activity. In the same studies, it was observed that, upon hydrolysis, the fractions derived from globulins showed a greater increase in antioxidant capacity, indicating that this property is related to the type of protein [42].

Sandoval-Sicairos et al. [43] reported higher antioxidant capacity values for flour and flour hydrolysates obtained with pepsin and pancreatin from *Amaranthus hypochondriacus* compared to those observed in the present study for proteins and peptides. These differences are likely due to the type of sample analyzed.

Phenols, tannins, flavonoids, and saponins are compounds reported in amaranth seeds and other plant parts, and are known to exhibit antioxidant activity [18,44]. Phenols were detected in CE and PF at significantly higher concentrations than those found in the peptides. Therefore, their presence may contribute to the free radical scavenging capacity observed in the samples analyzed.

It has been observed that metabolites such as phenols, condensed tannins, and anthocyanins may decrease upon hydrolysis of the samples, as was also found in this study for phenols. This reduction can be attributed to factors such as pH, the hydrolysis process, and the interactions of some of these compounds with proteins. Enzymatic hydrolysis of PF to obtain peptides may cause damage to the protein structure, resulting in the loss of the biological activity of the lectin. Consequently, no hemagglutinating effect was observed in the resulting peptides [43,45].

The cytotoxicity results of PF in both cell lines indicated a time- and dose-dependent effect. PF showed the highest cytotoxicity, followed by the P > 30 kDa, while P < 30 kDa showed the lowest cytotoxic effect, with the 72 h treatment causing the greatest cell damage.

Sabbione et al. [15] reported that the protein isolate and peptides obtained by pepsin and pancreatin digestion of *Amaranthus mantegazzianus* were able to inhibit the proliferation of colon cancer HT-29 cells, with a stronger inhibitory effect observed for the peptides (300 µg/mL) than for the protein isolate (1350 µg/mL). These results showed a higher cytotoxic effect on malignant cells compared to those obtained in the present study using MDA- MB-231 and SiHa cell lines.

The peptides derived from *A. hypochondriacus* exhibited lower cytotoxicity than those obtained by alcalase hydrolysis of *Amaranthus mantegazzianus* proteins, which were evaluated in osteosarcoma (MC3T3E1, UMR106) and colon cancer (Caco-2 and TC7) cell lines [4]. The differences observed between PF and peptides samples in this study may be attributed to the use of different *Amaranthus* species, variations in peptide production methods, particularly the enzymatic hydrolysis conditions applied, as well as the use of distinct cell lines in the cytotoxicity assays.

The protein extraction method employed in this study allowed the recovery of lectins, which were identified by their biological ability to agglutinate erythrocytes. Hemagglutinating activity was detected in both CE and PF, indicating that the extraction process did not impair the biological activity of these proteins.

The hemagglutinating activity was higher in PF, suggesting a greater abundance of lectins in this sample. Previous studies have also reported that the extraction method used here enables the isolation of lectins [46,47]. Therefore, the cytotoxic effect observed in PF could be attributed, at least in part, to the presence of lectins, as lectins from *A. caudatus*, *A. mantegazzianus*, and *A. hypochondriacus* have been shown to induce cytotoxicity and exert antiproliferative effects in cancer cell lines such as UMR106, CaCo-2, TC7, and MC3T3E1 [13,48].

It has been reported that protein extracts and protein concentrates from amaranth, common bean, and wheat, such as those prepared in the present study, exhibit toxic effects on normal and malignant cells, as well as in laboratory animals, causing both genotoxic and cytotoxic damage. These effects have been attributed to the presence of various metabolites, including bioactive peptides, lectins, phenols, saponins, and trypsin inhibitors contained in the samples [49,50].

In a previous study on crude extract from *Amaranthus hypochondriacus*, the presence of tannins, lectins, saponins, and trypsin inhibitors was reported. Furthermore, it exhibited genotoxic and cytotoxic effects on hepatic cells and erythrocytes [49]. Therefore, it is likely that PF obtained in the present study also contains tannins and trypsin inhibitors, which may contribute to the cytotoxic effects observed in the two analyzed cell lines.

The cytotoxic activity reported in the aforementioned studies differs from the results obtained in the present work for PF and peptide samples from *Amaranthus hypochondriacus*. These discrepancies may be attributed to the use of a different *Amaranthus* species, the evaluation of peptide samples with molecular weights above and below 30 kDa, and the use of different cell lines, all of which likely contributed to the observed variations in the results.

Cho et al. [51] reported that the wheat protein exhibited higher cytotoxic effect on breast cancer cell lines MCF-7 and MDA-MB-231 than that observed for PF in the present study. These authors reported a decrease in cell viability of 85.1% for MCF-7 and 87.5% for MDA-MB-231 after 72 h of treatment at a concentration of 100 µg/mL.

It is known that low-molecular-weight peptides exhibit greater cytotoxic activity against cancer cells [52,53]. However, higher-molecular-weight peptides, such as those derived from *Phaseolus coccineus*, have also been reported to exhibit greater bioactivity in MDA-MB-231 cells [31], which is consistent with the results obtained in the present study. Previous studies have shown that the production method, plant source, and amino acid sequence and composition of peptides can influence their biological interactions and mechanisms of action [54,55].

Another class of compounds that may contribute to membrane damage are saponins, which are amphiphilic glycosides known to interact strongly with membrane sterols. This interaction can lead to increased membrane permeability, pore formation, and ultimately membrane disruption [56,57]. Such effects are consistent with the ultrastructural damage induced by PF in the evaluated cell lines, including loss of membrane integrity and cellular morphology alterations. 

Although the saponin content in amaranth has been reported to be relatively low (0.9–4.9 mg/kg) [58], their biological impact cannot be excluded, especially considering that even low concentrations of these compounds may exert significant effects depending on their interaction with other molecules and the sensitivity of the target cells.

Importantly, the biological activity of saponins may be further enhanced by their interaction with other bioactive compounds present in the extract, such as lectins. Lectins are carbohydrate-binding proteins capable of recognizing specific glycosylated structures on the cell surface, which can facilitate cell membrane binding and internalization processes. This interaction may potentiate the effects of saponins by promoting their localization at the membrane level or by altering membrane organization, thereby increasing susceptibility to disruption [59].

In addition, lectins themselves have been reported to exert cytotoxic effects through mechanisms involving cell agglutination, membrane destabilization, and induction of apoptosis, particularly in cancer cells. Therefore, the combined presence of saponins and lectins in PF suggests a possible synergistic or complementary mechanism, contributing to the observed cytotoxicity and structural damage [60]. Thus, the observed cytotoxic effects of PF may result from the synergistic action of peptides, saponins, lectins, and other co-extracted compounds.

Phenolic compounds have been widely recognized for their potential anticancer properties, including pro-apoptotic, antiproliferative, anti-invasive, and cell cycle arrest activities, which are mediated through the modulation of multiple cellular signaling pathways involved in oxidative stress, inflammation, and apoptosis [61,62,63,64]. These biological effects are strongly influenced by their chemical structure, degree of hydroxylation, and interaction with cellular membranes and intracellular targets [63,64].

On the other hand, phenolic compounds may alter cancer cell physiology by regulating reactive oxygen species levels, mitochondrial function, and signaling pathways associated with cell survival and programmed cell death. Several studies have demonstrated that phenolic compounds can induce apoptosis through activation of caspase-dependent pathways and modulation of pro- and anti-apoptotic proteins [62,65].

Despite their reported cytotoxic potential at certain concentrations, polyphenols are also known for their cytoprotective effects. Their interaction with polar phospholipid groups contributes to membrane stabilization, reduction in lipid peroxidation, and preservation of cellular integrity under oxidative conditions [63,66]. Therefore, the biological effects of phenolic compounds may depend on their concentration, chemical composition, and the cellular context in which they act.

In the present study, the phenolic compounds detected in PF may have contributed, at least in part, to the observed biological activity. However, given their well-documented antioxidant and cytoprotective properties, further research is needed to investigate their specific contribution to membrane damage and cytotoxicity through the identification and quantification of individual phenolic metabolites.

According to the criteria established by the American National Cancer Institute, plant extracts are generally considered to exhibit significant cytotoxic potential when IC_50_ values are below 20 µg/mL (or 10 µM) after 48–72 h of incubation [67]. Based on these criteria, the IC_50_ values obtained in the present study indicate that none of the evaluated samples exhibited a relevant cytotoxic effect. However, peptides with molecular weights different from those evaluated in this study, as well as purified metabolites derived from amaranth, may exhibit greater biological activity.

Since PF caused the most significant cytotoxic damage in both cell lines after 72 h, it was selected for observation of the morphological changes induced in both cell lines. No morphological alterations were observed in the control, with cellular integrity maintained after 72 h. In contrast, cells treated with PF exhibited changes in membranes and organelles. The observed structural damage is likely associated with the mixture of compounds present in the sample, as each may individually induce cellular damage or, collectively, potentiate the disruption of membranes and organelles.

The cellular damage induced by PF is consistent with previous reports, which indicates that specific proteins, such as proteolytic enzymes and enzyme inhibitors, can alter membrane permeability [68]. Similarly, Uster and Pagano [69] reported that *Lens culinaris* lectin not only acts at the membrane level but can also internalize and interact with intracellular components. Additionally, *Viscum album* lectin has been demonstrated to induce membrane protrusions, resulting in apoptosis [70,71].

This study has several limitations, including the use of broad peptide fractionation, the absence of non-tumorigenic cell lines, and the lack of detailed molecular characterization of the bioactive components. Therefore, the results should be considered as preliminary screening.

Future studies should focus on molecular-level investigations to elucidate mechanisms of action and signaling pathways, as well as on the isolation and characterization of pure metabolites present in PF. In addition, it will be necessary to generate and evaluate lower molecular weight peptides, and to assess their biological activity in both cancer and non-cancerous cell lines. Finally, in vivo studies using appropriate animal models will be essential to validate the biological effects observed in vitro and to better establish the potential relevance of these compounds.

## 4. Materials and Methods

### 4.1. Plant Material

*Amaranthus hypochondriacus* seeds for this investigation were obtained from a producer in the municipality of Xochimilco, Mexico City. The seeds were cleaned and grounded in a grain mill (Analytical mill, 4301-00, Cole Parmer, Vernon Hills, IL, USA). The resulting flour was packed in airtight bags and refrigerated at 4 °C until its use.

### 4.2. Protein Extraction and Preparation of the Protein Fraction

Proteins were extracted from the amaranth flour with phosphate-buffered saline (PBS 1:10 *w*/*v*, pH 7.4) at 4 °C under constant agitation for 16 h. The mixture was centrifuged for 60 min at 10,000× *g* to remove insoluble solids; the supernatant, which corresponds to CE, was subjected to protein precipitation with 70% (NH_4_)_2_SO_4_ at 4 °C, and then centrifuged as previously indicated. The precipitate was resuspended, dialyzed overnight against H_2_O with three changes and lyophilized to obtain PF [46].

### 4.3. Enzymatic Hydrolysis

The method proposed by Teniente et al. [31] was followed. Briefly, sequential; enzymatic hydrolysis was carried out using pepsin (P7012, Sigma Chemical Co., St Louis, MO, USA) and pancreatin (P1750, Sigma Chemical Co., St Louis, MO, USA). PF was dissolved in distilled water (50 µg/mL) in a glass reactor, and the pH was adjusted to 2.0 with 1 N HCl at 37 °C; pepsin, previously dissolved in distilled water was added at an enzyme/substrate ratio of 1:25 and allowed to react for 60 min. Subsequently, the pH was adjusted to 5.3 by the addition of 0.9 M NaHCO_3_. Pancreatin solution was then added at an enzyme/substrate ratio of 1:20. The mixture was homogenized, the pH adjusted to 7.5 with 1 N NaOH, and incubated at 37 °C for 120 min. The hydrolysis reaction was stopped by heat treatment at 90 °C for 5 min. The resulting hydrolysate was stored at 4 °C for 24 h and then centrifuged at 12,000 rpm for 10 min.

### 4.4. Degree of Hydrolysis

DH was determined as the percentage of protein soluble in TCA, following the method described by Kim et al. [72]. Briefly, 10 mL aliquots of the hydrolysates were mixed with a 10% (*w*/*v*) TCA solution, allowed to stand for 10 min, and subsequently centrifuged at 12,000 rpm for 15 min. The N_2_ content of both the hydrolysates and the supernatants obtained after TCA treatment was determined using the Kjeldahl method [73]. DH was calculated using Equation (1):

%DH = (N_2_ soluble in 10% TCA/total N_2_ in the sample) × 100 (1)

where %DH represents the degree of hydrolysis, N_2_ corresponds to the nitrogen content of the sample, and TCA refers to trichloroacetic acid.

### 4.5. Peptide Fractionation by Ultrafiltration

Hydrolyzed proteins were fractionated by ultrafiltration with a stirred cell and disc membrane system (Amicon 8050 50 mL, Millipore, Burlington, MA, USA). Two peptide samples were separated from the protein hydrolysate using a cellulose membrane with a molecular weight cut-off (MWCO) of 30 kDa, to obtain P > 30 kDa and P < 30 kDa. Ultrafiltration was performed at room temperature (28 ± 2 °C) using O_2_ to maintain the transmembrane pressure at 75 psi. Once separation was complete, the samples were lyophilized and stored at 4 °C [31].

### 4.6. Protein Quantification

The protein content was quantified in the amaranth seeds according to the AOAC method (920.87), using a factor of 6.25 to calculate the total protein. The concentration of soluble proteins present in the CE, PF, and peptide samples was determined by the Bradford method using bovine serum albumin as the standard [74].

### 4.7. Antinutritional Compound Quantification

#### 4.7.1. Lectins

Human erythrocytes of blood type AB (+), obtained from healthy donors who signed an informed consent form, were used. The peripheral blood collection process was conducted in accordance with the official Mexican standards NOM-007-SSA3-2011 and NOM-253-SSA1-2012. Blood was collected using an anticoagulant, centrifuged for 10 min at 2000 rpm, washed three times with PBS and resuspended in PBS (2%).

The hemagglutination assay was carried out by the two-fold serial dilution method described by Valadez-Vega et al. [75] in 96-well U-bottom microtiter plates. Briefly, 50 μL of CE, PF and peptides solutions were added to the first well, and serial dilutions were performed by adjusting the volume of each well to 50 µL with PBS. Then, 50 µL of the 2% erythrocyte suspension was added to each well. The reaction mixture was incubated at room temperature for 60 min, after which the final dilution exhibiting visible hemagglutination was identified to determine the agglutination titer. The hemagglutination titer was expressed as the reciprocal of the highest dilution at which detectable agglutination was observed, corresponding to the inverse of the last dilution showing hemagglutination. HA/mg were calculated by dividing the hemagglutination titer by the soluble protein concentration in the sample, as determined by the Bradford method [74].

#### 4.7.2. Saponin Content

To determine these compounds, the methodology proposed by Valadez-Vega et al. [18] was used with some modifications. Briefly, the saponins were extracted from the four samples in methanol (10 mg/mL) under shaking for 30 min. The solvent was removed and the extracted saponins were dissolved in NaCl (0.9%).

To determine the hemolytic capacity of the saponins, the serial dilution method was employed using AB-type human erythrocytes. Erythrocytes were prepared as above and dissolved (2%) in NaCl (0.9%). Using a 96-well U-shaped microtiter plate, the saponin-containing solution was subjected to a twofold serial dilution. The volume of each sample in the wells was adjusted to 50 µL with NaCl (0.9%), and then 50 µL of a 4% erythrocyte suspension was added to each well. The reaction mixture was incubated for 1 h at room temperature, and the maximum dilution that demonstrated hemolysis was observed. The analyses were performed in triplicate, and the results were reported as HU/mg.

### 4.8. Total Phenolic Content

The quantification was carried out according to the Folin–Ciocalteu methodology as described by Singleton et al. [76]. All samples were dissolved in water (0.5 mg/mL) and shaken for 2 h at room temperature. The mixture was centrifuged for 15 min at 10,000 rpm and the resulting supernatant was used for quantification. To 100 μL of sample aliquot, 500 µL of Folin’s reagent (1:10) and 400 µL of 7.5% Na_2_CO_3_ were added. The mixture was then allowed to react at room temperature in darkness for 30 min and then the absorbance was measured at λ = 765 nm using a microplate reader (BioTek Epoch Instrument, Winooski, VT, USA). The results were reported in mg GAE/g, using gallic acid as the standard (Sigma Chemical Co., St Louis, MO, USA).

### 4.9. Antioxidant Capacity

#### 4.9.1. ABTS•+ Assay

The assay was performed according to the methodology described by Re et al. [77] using ABTS•+ (Sigma Chemical Co., St Louis, MO, USA) and 6-hydroxy-2,5,7,8-tetramethylchroman-2-carboxylic acid. (Trolox; Sigma Chemical Co., St Louis, MO, USA) as standard. ABTS•+ (3.6 mg/mL) was prepared 24 h before use at room temperature in darkness, in the presence of potassium persulfate (0.6 mg/mL; Sigma-Aldrich, St. Louis, MO, USA) in deionized water and then diluted in ethanol to obtain an absorbance of 0.70 ± 0.02 at λ = 734 nm. CE, PF and peptide samples were prepared in water (0.5 mg/mL), shaken for 2 h at room temperature, and then centrifuged for 15 min at 10,000 rpm. 900 µL of an ABTS•+ solution was added to 100 µL of each sample which was incubated in the dark at room temperature for 5 min, and then the absorbance was measured at λ = 734 nm. The results were expressed as mg TEAC/g.

#### 4.9.2. DPPH• Assay

The method proposed by Schenk and Brown [78] based on the reduction of DPPH• (Sigma Chemical Co., St Louis, MO, USA) was used. From CE, PF and peptide samples, 0.5 mg/mL dilutions of each sample were prepared in water and shaken for 2 h at room temperature. The mixture was centrifuged at 10,000 rpm for 15 min. To 100 µL of the supernatant, 900 µL of DPPH• solution (0.0075%) in ethanol was added, and the mixture was incubated in the darkness for 60 min and the absorbance was measured at λ = 540 nm. The results were expressed as mg TEAC/g using a calibration curve with Trolox as standard.

#### 4.9.3. Calculation of IC_50_ Values from Antioxidant Activity Assays

CE, PF, and peptide samples were prepared in distilled water at different concentrations ranging from 0 to 1 mg/mL. The ABTS•+ and DPPH• radicals were subsequently prepared as described in Section 4.9.1 and Section 4.9.2, respectively. Briefly, 100 µL of each sample concentration were mixed with 900 µL of the ABTS•+ or DPPH• solutions and incubated in darkness as indicated above. Absorbance measurements were then performed at λ = 734 and 540 nm, respectively.

IC_50_ values were determined by nonlinear regression analysis. Curve fitting was carried out using sigmoidal (Sigmoid) models, with coefficients of determination (R^2^) ranging from 0.95 to 0.98, and 95% confidence intervals.

### 4.10. Cytotoxicity Assay

A cytotoxicity assay was performed using the human malignant cell lines MDA-MB-231 (triple-negative breast adenocarcinoma cells of epithelial origin, derived from a 51-year-old Caucasian female) and SiHa (human cervical squamous carcinoma cells of epithelial origin, derived from a 55-year-old Asian female and containing integrated copies of human papillomavirus type 16); both cell lines were obtained from the American Type Culture Collection (ATCC). The cells were cultured in DMEM (Gibco, Grand Island, NY, USA) with 10% FBS and 0.1% antibiotic (a combination of streptomycin and penicillin; Gibco) at 37 °C in a CO_2_ water-jacketed incubator (Sanyo, Osaka, Japan), in a humidified atmosphere containing 5% CO_2_ and 95% air.

The analysis was performed using the colorimetric method described by Valadez-Vega et al. [79] focusing on the mitochondrial function of the treated cells. The MTT (3-(4,5-dimethylthiazol-2-yl)-2-5-diphenyltetrazolium bromide) assay is a colorimetric test that uses the tetrazolium dye to assess cell viability. The MDA-MB-231 and SiHa cell lines were cultured in 96-well microplates at 10,000 cells/well for the 24 h assay, and 5000 cells/well for the 48 and 72 h assays. After 24 h the culture medium was replaced with a new medium containing the samples; for PF the concentrations were between 0–2500 mg/mL, P > 30 kDa were 0–1000 mg/mL, P < 30 kDa 0–3000 mg/mL, and CDDP 0–0.1 mg/mL, in both cell lines, the cytotoxicity assays were carried out for 24, 48 and 72 h.

After the cells were exposed, the sample solution was replaced by MTT reagent (5 mg/mL), and the plate was incubated for 3 h at 37 °C. The media was removed, and the intracellular formazan product was dissolved in dimethyl sulfoxide (DMSO). Absorbance was measured at λ = 540 nm (BioTek Epoch instrument) and cell viability was calculated relative to the blank (100% viability).

#### Calculation of IC_50_ Values from Cytotoxicity Assays

IC_50_ values were determined by nonlinear regression analysis of dose–response curves obtained from cytotoxicity assays. Curve fitting was performed using Sigmoidal (Hill and Sigmoid), Exponential Decay (Triple) and Peak (Pseudo-Voigt) models, with R^2^ ranging from 0.90 to 0.99, and a 95% confidence interval.

### 4.11. Transmission Electron Microscopy 

For the ultrastructure analysis, MDA-MB-231 and SiHa cells were treated for 72 h with the PF at concentration of 44.98 µg/mL and 69.24 µg/mL, respectively. The cells were collected and fixed with 2.5% glutaraldehyde. Morphological changes in cells were observed using a JEM1200EXII transmission electron microscope (JEOL, Tokyo, Japan).

### 4.12. Statistical Analysis

Quantitative data is expressed as the mean  ±  SD, and analysis of variance (ANOVA) was performed, followed by Tukey post hoc test (*p* ≤ 0.05). StatGraphics Centurion version 19 (StatGraphics Technologies, Inc., The Plains, VA, USA) software was used for data analysis, while SigmaPlot version 12.3 (Systat Software Inc., San Jose, CA, USA) was employed for IC_50_ value calculations; all experimental determinations were performed in triplicate.

## 5. Conclusions

All amaranth-derived samples contained phenolic compounds, while CE and PF additionally exhibited the presence of saponins and lectins. All samples showed antioxidant capacity, which decreased during the peptide production process. All samples exerted in vitro cytotoxic effects in both evaluated cell lines. Among them, PF displayed the highest biological activity after 72 h of exposure, particularly in MDA-MB-231 cells; however, other co-extracted metabolites may contribute to the observed bioactivity. TEM analysis revealed that PF induced significant structural damage in both cell lines after 72 h.

It is important to note that all amaranth-derived samples showed high IC_50_ values at all evaluated time points, indicating limited pharmacological relevance under standard anticancer criteria. However, peptides with different molecular weights and purified amaranth metabolites may exhibit greater biological activity.

Future studies should focus on elucidating the molecular mechanisms involved, isolating and characterizing bioactive metabolites, and evaluating peptides of different molecular weight in both cancerous and non-cancerous cell lines, as well as in vivo models to validate their biological activity.

## Figures and Tables

**Table 1:** Phenolic and antinutritional compound content in amaranth samples.

Sample	Total Phenols	Saponins	Lectins
	(mg GAE/g)	(HU/mg)	(HA/mg)
CE	129.40 ± 2.4 ^a^	23.81 ± 0 ^a^	7.00 ± 0.1 ^a^
PF	28.01 ± 0.03 ^b^	80.00 ± 0 ^b^	60.30 ± 0.1 ^b^
P > 30 kDa	11.78 ± 0.08 ^c^	ND	ND
P < 30 kDa	11.49 ± 0.02 ^c^	ND	ND

Crude extract (CE), protein fraction (PF), peptides > 30 kDa (P > 30 kDa), peptides < 30 kDa (P < 30 kDa), mg of gallic acid equivalents/g (mg GAE/g), hemolytic units/mg (HU/mg), hemagglutination activity/mg (HA/mg). Values represent the mean of three independent replicates ± SD. Letters ^a^, ^b^, ^c^ indicate statistically significant differences among samples found in Tukey post hoc tests (*p* ≤ 0.05). ND—no data.

**Table 2:** Antioxidant capacity and IC_50_.

Sample	ABTS•+		DPPH•	
	µM TEAC/g	IC_50_ (µM/mL)	µM TEAC/g	IC_50_ (µM/mL)
CE	104.50 ± 0.69 ^a^	7.10 ± 0.30 ^a^	75.62 ± 0.21 ^a^	5.15 ± 0.20 ^a^
PF	19.40 ± 0.05 ^b^	9.82 ± 0.30 ^b^	17.10 ± 0.14 ^b^	11.39 ± 1.60 ^b^
P > 30 kDa	9.82 ± 0.10 ^c^	136.40 ± 0.90 ^c^	9.42 ± 0.04 ^c^	165.19 ± 4.20 ^c^
P < 30 kDa	8.04 ± 0.05 ^d^	105.53 ± 0.10 ^d^	12.30 ± 0.26 ^d^	150.90 ± 7.90 ^d^

Crude extract (CE), protein fraction (PF), peptides > 30 kDa (P > 30 kDa), peptides < 30 kDa (P < 30 kDa), µM of Trolox equivalent antioxidant capacity/g (µM TEAC/g), mean inhibitory concentration (IC_50_). Data shown represent the mean ± SD obtained from three independent experiments. Letters ^a^, ^b^, ^c^, ^d^ indicate statistically significant differences among samples found in Tukey post hoc tests (*p* ≤ 0.05).

**Table 3:** IC_50_ of amaranth samples against MDA-MB-231 and SiHa cell lines.

		IC_50_ (mg/mL)			
		PF	P > 30 kDa	P < 30 kDa	CDDP
MDA-MB-231	24 h	401.44 ± 23.24 ^a^_w_	783.31 ± 12.99 ^a^_x_	2362.92 ± 36.92 ^a^_y_	0.091 ± 0.000 ^a^_z_
	48 h	123.66 ± 18.65 ^b^_w_	880.77 ± 6.60 ^b^_x_	1823.62 ± 6.33 ^b^_y_	0.013 ± 0.0001 ^b^_z_
	72 h	44.98 ± 1.21 ^c^_w_	875.74 ± 4.92 ^b^_x_	1595.51 ± 22.02 ^c^_y_	0.008 ± 0.000 ^c^_z_
SiHa	24 h	401.30 ± 22.93 ^a^_w_	854.83 ± 12.62 ^ab^_x_	>3000 ^a^_z_	0.058 ± 0.0002 ^a^_z_
	48 h	744.44 ± 19.24 ^b^_w_	873.33 ± 7.38 ^a^_x_	1681.64 ± 30.96 ^b^_y_	0.019 ± 0.0002 ^b^_z_
	72 h	69.24 ± 8.06 ^c^_w_	836.57 ± 11.83 ^b^_x_	1470.43 ± 13.91 ^c^_y_	0.006 ± 0.0001 ^c^_z_

Protein fraction (PF), peptides > 30 kDa (P > 30 kDa), peptides < 30 kDa (P < 30 kDa), cisplatin (CDDP), mean inhibitory concentration (IC_50_). Values represent the mean ± SD obtained from three independent experiments. Letters ^a^, ^b^, ^c^ indicate statistically significant differences among exposure times, letters _w_, _x_, _y_, _z_ indicate differences among samples, found in Tukey post hoc tests (*p* ≤ 0.05).

## Data Availability

Data is contained within this article.

## Abbreviations

The following abbreviations are used in this manuscript:

ABTS•+	2,2′-Azino-bis(3-ethylbenzothiazoline-6-sulfonic acid)
ANOVA	Analysis of variance
ATCC	American Type Culture Collection
CDDP	Cisplatin
CE	Crude extract
DH	Degree of hydrolysis
DMEM	Dulbecco’s modified Eagle’s medium
DMSO	Dimethyl sulfoxide
DPPH•	2,2-diphenyl-1-picrylhydrazyl
FBS	Fetal bovine serum
GAE	Gallic acid equivalents
HA	Hemagglutination activity
HU	Hemolytic units
IC_50_	Mean inhibitory concentration
MTT	3-(4,5-dimethylthiazol-2-yl)-2-5-diphenyltetrazolium bromide
P < 30 kDa	Peptides < 30 kDa
P > 30 kDa	Peptides > 30 kDa
PF	Protein fraction
R^2^	Coefficient of determination
SD	Standard deviation
TCA	Trichloroacetic acid
TEAC	Trolox equivalent antioxidant capacity
TEM	Transmission electron microscopy
Trolox	6-hydroxy-2,5,7,8-tetramethylchroman-2-carboxylic acid

## References

[1] Cancer Today. https://gco.iarc.fr/today.

[2] Hanahan D. (2022). Hallmarks of Cancer: New Dimensions. Cancer Discov..

[3] Greenwell M., Rahman P.K.S.M. (2015). Medicinal Plants: Their Use in Anticancer Treatment. Int. J. Pharm. Sci. Res..

[4] Barrio D.A., Añón M.C. (2010). Potential Antitumor Properties of a Protein Isolate Obtained from the Seeds of *Amaranthus mantegazzianus*. Eur. J. Nutr..

[5] Loveday S.M. (2019). Food Proteins: Technological, Nutritional, and Sustainability Attributes of Traditional and Emerging Proteins. Annu. Rev. Food Sci. Technol..

[6] Romero-Benavides J.C., Guaraca-Pino E., Duarte-Casar R., Rojas-Le-Fort M., Bailon-Moscoso N. (2023). *Chenopodium quinoa* Willd. and *Amaranthus hybridus* L.: Ancestral Andean Food Security and Modern Anticancer and Antimicrobial Activity. Pharmaceuticals.

[7] Caselato-Sousa V.M., Amaya-Farfán J. (2012). State of Knowledge on Amaranth Grain: A Comprehensive Review. J. Food Sci..

[8] Aderibigbe O.R., Ezekiel O.O., Owolade S.O., Korese J.K., Sturm B., Hensel O. (2022). Exploring the Potentials of Underutilized Grain Amaranth (*Amaranthus* spp.) along the Value Chain for Food and Nutrition Security: A Review. Crit. Rev. Food Sci. Nutr..

[9] Toimbayeva D., Saduakhasova S., Kamanova S., Kiykbay A., Tazhina S., Temirova I., Muratkhan M., Shaimenova B., Murat L., Khamitova D. (2025). Prospects for the Use of Amaranth Grain in the Production of Functional and Specialized Food Products. Foods.

[10] Master P.B.Z., Macedo R.C.O. (2021). Effects of Dietary Supplementation in Sport and Exercise: A Review of Evidence on Milk Proteins and Amino Acids. Crit. Rev. Food Sci. Nutr..

[11] Calderón de la Barca A.M., Mercado-Gómez L.E., Heredia-Sandoval N.G., Luna-Alcocer V., Loaiza P.M.A.P., González-Ríos H., Islas-Rubio A.R. (2022). Highly Nutritional Bread with Partial Replacement of Wheat by Amaranth and Orange Sweet Potato. Foods.

[12] Udenigwe C.C., Aluko R.E. (2012). Food Protein-Derived Bioactive Peptides: Production, Processing, and Potential Health Benefits. J. Food Sci..

[13] Quiroga A.V., Barrio D.A., Añón M.C. (2015). Amaranth Lectin Presents Potential Antitumor Properties. LWT—Food Sci. Technol..

[14] Lado M.B., Burini J., Rinaldi G., Añón M.C., Tironi V.A. (2015). Effects of the Dietary Addition of Amaranth (*Amaranthus mantegazzianus*) Protein Isolate on Antioxidant Status, Lipid Profiles and Blood Pressure of Rats. Plant Foods Hum. Nutr..

[15] Sabbione A.C., Ogutu F.O., Scilingo A., Zhang M., Añón M.C., Mu T.H. (2019). Antiproliferative Effect of Amaranth Proteins and Peptides on HT-29 Human Colon Tumor Cell Line. Plant Foods Hum. Nutr..

[16] Baraniak J., Kania-Dobrowolska M. (2022). The Dual Nature of Amaranth—Functional Food and Potential Medicine. Foods.

[17] Korhonen H., Pihlanto A. (2006). Bioactive Peptides: Production and Functionality. Int. Dairy J..

[18] Valadez-Vega C., Lugo-Magaña O., Figueroa-Hernández C., Bautista M., Betanzos-Cabrera G., Bernardino-Nicanor A., González-Amaro R.M., Alonso-Villegas R., Morales-González J.A., González-Cruz L. (2022). Effects of Germination and Popping on the Anti-Nutritional Compounds and the Digestibility of *Amaranthus hypochondriacus* Seeds. Foods.

[19] Shahbaz M., Raza N., Islam M., Imran M., Ahmad I., Meyyazhagan A., Pushparaj K., Balasubramanian B., Park S., Rengasamy K.R.R. (2023). The Nutraceutical Properties and Health Benefits of Pseudocereals: A Comprehensive Treatise. Crit. Rev. Food Sci. Nutr..

[20] Jan N., Hussain S.Z., Naseer B., Bhat T.A. (2023). Amaranth and Quinoa as Potential Nutraceuticals: A Review of Anti-Nutritional Factors, Health Benefits and Their Applications in Food, Medicinal and Cosmetic Sectors. Food Chem. X.

[21] Venskutonis P.R., Kraujalis P. (2013). Nutritional Components of Amaranth Seeds and Vegetables: A Review on Composition, Properties, and Uses. Compr. Rev. Food Sci. Food Saf..

[22] López D.N., Galante M., Robson M., Boeris V., Spelzini D. (2018). Amaranth, Quinoa and Chia Protein Isolates: Physicochemical and Structural Properties. Int. J. Biol. Macromol..

[23] Alu’datt M.H., Rababah T., Alhamad M.N., Alodat M., Al-Mahasneh M.A., Gammoh S., Ereifej K., Almajwal A., Kubow S. (2017). Molecular Characterization and Bio-Functional Property Determination Using SDS-PAGE and RP-HPLC of Protein Fractions from Two *Nigella* Species. Food Chem..

[24] Singh J., Kamboj K.K., Kamboj S.S., Sandhu R.S., Shangary S. (1993). Affinity Purification and Characterization of Lectins from Two *Amaranthus* Species. Plant Sci..

[25] Hernández de la Torre M., Covaleda-Cortés G., Montesinos L., Covaleda D., Ortiz J.C., Piñol J., Bautista J.M., Castillo J.P., Reverter D., Avilés F.X. (2025). Analysis of Protein Inhibitors of Trypsin in Quinoa, Amaranth and Lupine Seeds. Selection and Deep Structure–Function Characterization of the *Amaranthus caudatus* Species. Int. J. Mol. Sci..

[26] Avanza M.V., Añón M.C. (2007). Effect of Thermal Treatment on the Proteins of Amaranth Isolates. J. Sci. Food Agric..

[27] Thanapornpoonpong S.N., Vearasilp S., Pawelzik E., Gorinstein S. (2008). Influence of Various Nitrogen Applications on Protein and Amino Acid Profiles of Amaranth and Quinoa. J. Agric. Food Chem..

[28] Huerta-Ocampo J.Á., de la Rosa A.P.B. (2011). Amaranth: A Pseudo-Cereal with Nutraceutical Properties. Curr. Nutr. Food Sci..

[29] Montoya-Rodríguez A., Milán-Carrillo J., Reyes-Moreno C., de Mejía E.G. (2015). Characterization of Peptides Found in Unprocessed and Extruded Amaranth (*Amaranthus hypochondriacus*) Pepsin/Pancreatin Hydrolysates. Int. J. Mol. Sci..

[30] Mora-Escobedo R., Robles-Ramírez M.D.C., Ramón-Gallegos E., Reza-Alemán R. (2009). Effect of Protein Hydrolysates from Germinated Soybean on Cancerous Cells of the Human Cervix: An In Vitro Study. Plant Foods Hum. Nutr..

[31] Teniente-Martínez G., Bernardino-Nicanor A., Cariño-Cortés R., Valadez-Vega M.D.C., Montañez-Soto J.L., Acosta-García G., González-Cruz L. (2019). Cytotoxic and Genotoxic Activity of Protein Isolate of Ayocote Beans and Anticancer Activity of Their Protein Fractions. J. Food Meas. Charact..

[32] Ayala-Niño A., Rodríguez-Serrano G.M., Jiménez-Alvarado R., Bautista-Avila M., Sánchez-Franco J.A., González-Olivares L.G., Cepeda-Saez A. (2019). Bioactivity of Peptides Released during Lactic Fermentation of Amaranth Proteins with Potential Cardiovascular Protective Effect: An In vitro Study. J. Med. Food.

[33] Sabbione A.C., Scilingo A., Añón M.C. (2015). Potential Antithrombotic Activity Detected in Amaranth Proteins and Its Hydrolysates. LWT—Food Sci. Technol..

[34] Peña-Ramos E.A., Xiong Y.L., Arteaga G.E. (2004). Fractionation and Characterisation for Antioxidant Activity of Hydrolysed Whey Protein. J. Sci. Food Agric..

[35] Aluko R.E., Shahidi F. (2015). Amino Acids, Peptides, and Proteins as Antioxidants for Food Preservation. Handbook of Antioxidants for Food Preservation.

[36] Sarmadi B.H., Ismail A. (2010). Antioxidative Peptides from Food Proteins: A Review. Peptides.

[37] Najafian L., Babji A.S. (2015). Isolation, Purification and Identification of Three Novel Antioxidative Peptides from Patin (*Pangasius sutchi*) Myofibrillar Protein Hydrolysates. LWT—Food Sci. Technol..

[38] Zou T.B., He T.P., Li H.B., Tang H.W., Xia E.Q. (2016). The Structure-Activity Relationship of the Antioxidant Peptides from Natural Proteins. Molecules.

[39] Jo H.J., Chung K.H., Yoon J.A., Lee K.J., Song B.C., An J.H. (2015). Radical Scavenging Activities of Tannin Extracted from Amaranth (*Amaranthus caudatus* L.). J. Microbiol. Biotechnol..

[40] Ayala-Niño A., Rodríguez-Serrano G.M., González-Olivares L.G., Contreras-López E., Regal-López P., Cepeda-Saez A. (2019). Sequence Identification of Bioactive Peptides from Amaranth Seed Proteins (*Amaranthus hypochondriacus* spp.). Molecules.

[41] Orsini Delgado M.C., Galleano M., Añón M.C., Tironi V.A. (2015). Amaranth Peptides from Simulated Gastrointestinal Digestion: Antioxidant Activity Against Reactive Species. Plant Foods Hum. Nutr..

[42] Capraro J., De Benedetti S., Heinzl G.C., Scarafoni A., Magni C. (2021). Bioactivities of Pseudocereal Fractionated Seed Proteins and Derived Peptides Relevant for Maintaining Human Well-Being. Int. J. Mol. Sci..

[43] Sandoval-Sicairos E.S., Domínguez-Rodríguez M., Montoya-Rodríguez A., Milán-Noris A.K., Reyes-Moreno C., Milán-Carrillo J. (2020). Phytochemical Compounds and Antioxidant Activity Modified by Germination and Hydrolysis in Mexican Amaranth. Plant Foods Hum. Nutr..

[44] Kongdang P., Dukaew N., Pruksakorn D., Koonrungsesomboon N. (2021). Biochemistry of *Amaranthus* Polyphenols and Their Potential Benefits on Gut Ecosystem: A Comprehensive Review of the Literature. J. Ethnopharmacol..

[45] Acosta-Estrada B.A., Gutiérrez-Uribe J.A., Serna-Saldívar S.O. (2014). Bound Phenolics in Foods, a Review. Food Chem..

[46] Valadez-Vega C., Lugo-Magaña O., Betanzos-Cabrera G., Villagómez-Ibarra J.R. (2022). Partial Characterization of Lectins Purified from the Surco and Vara (Furrow and Rod) Varieties of Black *Phaseolus vulgaris*. Molecules.

[47] Velhal M.K., Shenoy V.S., Upadhye V., Sinha R.P. (2023). Extraction and Purification of Lectin from Soybean Seeds (Glycine Max). Lett. Appl. NanoBioSci..

[48] Mengoni A., Quiroga A.V., Añón M.C. (2016). Purificación y Caracterización de Una Lectina de *Amaranthus hypochondriacus*, Un Compuesto Antiproliferativo. INNOTEC.

[49] Valadez-Vega C., Lugo-Magaña O., Morales-González J.A., Delgado-Olivares L., Izquierdo-Vega J.A., Sánchez-Gutiérrez M., López-Contreras L., Bautista M., Velázquez-González C. (2021). Phytochemical, Cytotoxic, and Genotoxic Evaluation of Protein Extract of *Amaranthus hypochondriacus* Seeds. CyTA-J. Food.

[50] Vasconcelos Severino Q.D.J., Bachur Rodrigues T.P., Frota Aragão G. (2021). Whey Protein Supplementation and Its Potentially Adverse Effects on Health: A Systematic Review. Appl. Physiol. Nutr. Metab..

[51] Cho K., Lee C.W., Ohm J.B. (2016). In Vitro Study on Effect of Germinated Wheat on Human Breast Cancer Cells. Cereal Chem..

[52] Quintal-Bojórquez N., Segura-Campos M.R. (2021). Bioactive Peptides as Therapeutic Adjuvants for Cancer. Nutr. Cancer.

[53] Luna Vital D.A., González De Mejía E., Dia V.P., Loarca-Piña G. (2014). Peptides in Common Bean Fractions Inhibit Human Colorectal Cancer Cells. Food Chem..

[54] Avilés-Gaxiola S., García-Aguiar I., Jiménez-Ortega L.A., Gutiérrez-Grijalva E.P., Heredia J.B. (2025). Bioactive Plant Peptides: Physicochemical Features, Structure-Function Insights and Mechanism of Action. Molecules.

[55] Rayaprolu S.J., Hettiarachchy N.S., Horax R., Kumar-Phillips G., Liyanage R., Lay J., Chen P. (2017). Purification and Characterization of a Peptide from Soybean with Cancer Cell Proliferation Inhibition. J. Food Biochem..

[56] Carpinteyro Díaz A.E., Herfindal L., Rathe B.A., Sletta K.Y., Vedeler A., Haavik S., Fossen T. (2019). Cytotoxic Saponins and Other Natural Products from Flowering Tops of *Narthecium ossifragum* L. Phytochemistry.

[57] Lorent J.H., Quetin-Leclercq J., Mingeot-Leclercq M.P. (2014). The Amphiphilic Nature of Saponins and Their Effects on Artificial and Biological Membranes and Potential Consequences for Red Blood and Cancer Cells. Org. Biomol. Chem..

[58] Aguirre-García Y.L., Castillo-Manzanares A., Palomo-Ligas L., Ascacio-Valdés J.A., Campos-Múzquiz L.G., Esparza-González S.C., Rodríguez-Herrera R., Nery-Flores S.D. (2024). Toxicity Evaluation of a Polyphenolic Extract from *Flourensia cernua* DC through *Artemia* Lethality Assay, Hemolytic Activity, and Acute Oral Test. J. Toxicol..

[59] Yau T., Dan X., Ng C.C.W., Ng T.B. (2015). Lectins with Potential for Anti-Cancer Therapy. Molecules.

[60] Mazalovska M., Kouokam J.C. (2020). Plant-Derived Lectins as Potential Cancer Therapeutics and Diagnostic Tools. BioMed Res. Int..

[61] Cory H., Passarelli S., Szeto J., Tamez M., Mattei J. (2018). The Role of Polyphenols in Human Health and Food Systems: A Mini-Review. Front. Nutr..

[62] Hazafa A., Rehman K.U., Jahan N., Jabeen Z. (2020). The Role of Polyphenol (Flavonoids) Compounds in the Treatment of Cancer Cells. Nutr. Cancer.

[63] Fraga C.G., Croft K.D., Kennedy D.O., Tomás-Barberán F.A. (2019). The Effects of Polyphenols and Other Bioactives on Human Health. Food Funct..

[64] Maleki Dana P., Sadoughi F., Asemi Z., Yousefi B. (2022). The Role of Polyphenols in Overcoming Cancer Drug Resistance: A Comprehensive Review. Cell. Mol. Biol. Lett..

[65] Bhosale P.B., Ha S.E., Vetrivel P., Kim H.H., Kim S.M., Kim G.S. (2020). Functions of Polyphenols and Its Anticancer Properties in Biomedical Research: A Narrative Review. Transl. Cancer Res..

[66] Schewe T., Kühn H., Sies H. (2002). Flavonoids of Cocoa Inhibit Recombinant Human 5-Lipoxygenase. J. Nutr..

[67] Virchea L.I., Frum A., Georgescu C., Pecsenye B., Máthé E., Mironescu M., Crăciunaș M.T., Totan M., Tănăsescu C., Gligor F.G. (2025). An Overview of the Bioactivity of Spontaneous Medicinal Plants Suitable for the Improvement of Lung Cancer Therapies. Pharmaceutics.

[68] Ramos M.V., Brito D., Freitas C.D.T., Gonçalves J.F.C., Porfirio C.T.M.N., Lobo M.D.P., Monteiro-Moreira A.C.O., Souza L.A.C., Fernandes A.V. (2018). Proteomic Identification and Purification of Seed Proteins from Native Amazonian Species Displaying Antifungal Activity. Planta.

[69] Uster P.S., Pagano R.E. (1986). Resonance Energy Transfer Microscopy: Observations of Membrane-Bound Fluorescent Probes in Model Membranes and in Living Cells. J. Cell Biol..

[70] Büssing A. (1996). Induction of Apoptosis by the Mistletoe Lectins: A Review on the Mechanisms of Cytotoxicity Mediated by *Viscum album* L. Apoptosis.

[71] Büssing A., Suzart K., Bergmann J., Pfüller U., Schietzel M., Schweizer K. (1996). Induction of Apoptosis in Human Lymphocytes Treated with *Viscum album* L. Is Mediated by the Mistletoe Lectins. Cancer Lett..

[72] Kim S.Y., Park P.S.W., Rhee K.C. (1990). Functional Properties of Proteolytic Enzyme Modified Soy Protein Isolate. J. Agric. Food Chem..

[73] Cunniff P. (1999). Official Methods of Analysis of AOAC International.

[74] Kruger N.J., Walker J.M. (2009). The Bradford Method for Protein Quantitation. The Protein Protocols Handbook.

[75] Valadez-Vega C., Álvarez-Manilla G., Riverón-Negrete L., García-Carrancá A., Morales-González J.A., Zuniga-Pérez C., Madrigal-Santillán E., Esquivel-Soto J., Esquivel-Chirino C., Villagómez-Ibarra R. (2011). Detection of Cytotoxic Activity of Lectin on Human Colon Adenocarcinoma (Sw480) and Epithelial Cervical Carcinoma (C33-A). Molecules.

[76] Singleton V.L., Rossi J.A. (1965). Colorimetry of Total Phenolics with Phosphomolybdic-Phosphotungstic Acid Reagents. Am. J. Enol. Vitic..

[77] Re R., Nicoletta P., Anna P., Ananth P., Min Y., Catherine R.-E. (1999). Antioxidant Activity Applying an Improved ABTS Radical Cation Decolorization Assay. Free Radic. Biol. Med..

[78] Schenk G.H., Brown D.J. (1967). Free Radical Oxidation of Dihydric Phenols with Diphenylpicrylhydrazyl. Talanta.

[79] Valadez-Vega C., Lugo-Magaña O., Mendoza-Guzmán L., Villagómez-Ibarra J.R., Velasco-Azorsa R., Bautista M., Betanzos-Cabrera G., Morales-González J.A., Madrigal-Santillán E.O. (2023). Antioxidant Activity and Anticarcinogenic Effect of Extracts from *Bouvardia ternifolia* (Cav.) Schltdl. Life.

